# Nurses’ scope of practice and fundamental care in relation to older people: An exploratory home-based study

**DOI:** 10.1016/j.ijnsa.2026.100492

**Published:** 2026-01-26

**Authors:** Karin Sandberg, Anna Josse Eklund, Gunilla Borglin, Edith Roth Gjevjon, Cecilia Olsson

**Affiliations:** a Department of Health Sciences, Faculty of Health, Science, and Technology, Karlstad University, Karlstad, Sweden; b Department of Bachelor’s Education (Nursing), Lovisenberg Diaconal University College, Oslo, Norway

**Keywords:** Fundamental care, Registered nurses, Non-registered nurses, Home care, Nursing process, Decision-making

## Abstract

**Background:**

Fundamental care—encompassing relational, psychosocial, and physical needs as outlined in the Fundamentals of Care Framework—is a multifaceted yet essential component of nurses’ scope of practice. Despite its significance, fundamental care remains underrepresented in research within home-based care. Consequently, limited knowledge exists regarding how nurses address older people's fundamental care needs, the practical applicability of the Framework, and the influence of contextual modulators.

**Aim:**

To explore nurses’ scope of practice, fundamental care, and contextual modulators in relation to older people with complex health care needs in home-based care.

**Design:**

Exploratory design.

**Setting:**

Four home-care sites in Western Sweden.

**Methods:**

Structured direct observations were conducted using a protocol informed by the Fundamentals of Care Framework and concepts relevant to nurses’ scope of practice. Quantitative data were analysed using descriptive statistics and contextualised by qualitative field notes.

**Result:**

A total of 3042 care activities were recorded across 230 observations involving 46 nurses (registered and non-registered). On average, participants performed 13.23 activities per observation, often addressing multiple dimensions of the Fundamentals of Care Framework. Physical needs typically served as the entry point for care, which frequently expanded to include relational and psychosocial aspects. Registered nurses’ involvement in clinical decision-making—structured around the five phases of the nursing process—was primarily concentrated on assessment and implementation. Non-registered nurses also engaged in decision-making and independently initiated activities. Nurses’ scope of practice appeared to be related to several contextual modulators, including interruptions and a lack of supportive work environments.

**Conclusion and implications:**

We are among the first to explore nurses’ scope of practice in home-based care using the Fundamentals of Care Framework as a conceptual foundation. We have underscored the complexity and multifaceted nature of nurses’ scope of practice, including clinical decision-making, the activities’ functional and performance levels, and the presence of contextual modulators. Task-shifting from registered nurses to non-registered nurses was evident in clinical decision-making. We suggest that future Models of Care grounded in the Framework and tailored to the specific contextual conditions of home-based care may support nurses in delivering high-quality fundamental care.

## Abstract (author highlights)

• Researchers have indicated that fundamental care is sometimes inadequately delivered, deprioritized, or omitted—leading to increased risks of adverse events, including mortality.
• Fundamental care constitutes a particularly important aspect of nurses’ scope of practice in home-based care, given the complex health care needs of older people.
• Critical thinking and active engagement in clinical decision-making form a cornerstone of registered nurses’ scope of practice.
• Participants simultaneously engaged in activities related to the nurse–patient relationship and the integration of care—including its relational, psychosocial, and physical sub-dimensions—highlighting the complexity of nurses’ scope of practice in home-based care.
• Both registered nurses and non-registered nurses were involved in clinical decision-making, underscoring the autonomy of non-registered nurses and indicating ongoing task-shifting.
• From our observations of contextual modulators, we suggest that nurses’ scope of practice may be affected by an unsupportive working environment and frequent interruptions during home visits.

**What is already known**

• Researchers have indicated that fundamental care is sometimes inadequately delivered, deprioritized, or omitted-leading to increased risks of adverse events, including mortality.
• Fundamental care constitutes a particularly important aspect of nurses' scope of practice in home-based care, given the complex health care needs of older people.
• Critical thinking and active engagement in clinical decision-making form a cornerstone of registered nurses... scope of practice.

Alt-text: Unlabelled box dummy alt text

**What this paper adds**

• Participants simultaneously engaged in activities related to the nurse...patient relationship and the integration of care-including its relational, psychosocial, and physical sub-dimensions-highlighting the complexity of nurses' scope of practice in home-based care.
• Both registered nurses and non-registered nurses were involved in clinical decision-making, underscoring the autonomy of non-registered nurses and indicating ongoing task-shifting.
• From our observations of contextual modulators, we suggest that nurses... scope of practice may be affected by an unsupportive working environment and frequent interruptions during home visits.

Alt-text: Unlabelled box dummy alt text

## 1 Background

**Fig. 1:**
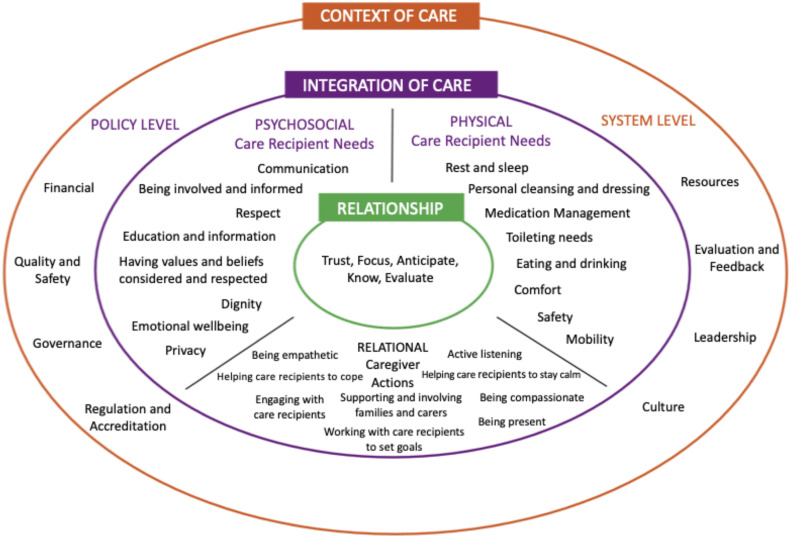
The Fundamentals of Care Framework. Obtained from the International Learning Collaborative (n.d). Content within image derived from Kitson et al. (2013) and Feo et al. (2018). Reprinted with permission.

The primary focus within nurses’ scope of practice is to ensure the provision of fundamental care (Henderson, 1964) and to optimize health outcomes (Nightingale, 1992). However, researchers have indicated that fundamental care is at times inadequately delivered, deprioritized, or even omitted—resulting in increased risks of adverse events, including mortality (Aiken et al., 2017; Kalánková et al., 2021). This has raised concerns about the extent to which nurses assume responsibility for meeting older people’s fundamental care needs and about the degree to which healthcare organizations support nurses in fulfilling these responsibilities (Kalánková et al., 2021; Sworn and Booth, 2020). Furthermore, in the context of fundamental care, nurses have been found to rely on habitual routines and intuitive judgments rather than on evidence-based theoretical frameworks (Pavedahl et al., 2024; Richards and Borglin, 2019). In response to this gap, Kitson and colleagues developed the Fundamentals of Care framework (Fig. 1), hereafter referred to as “the Framework”, to guide nurses in delivering high-quality fundamental care, that integrates relational, psychosocial, and physical care needs (Feo et al., 2018; Kitson et al., 2013, 2010).

For nurses—here referring to both RNs (with a bachelor’s degree) and non-RNs (such as nursing assistants and care assistants)—the delivery of fundamental care begins with establishing a nurse–patient relationship through an individualized approach. This involves building and maintaining trust, engaging attentively, and developing an in-depth understanding of the patient. Central to this process are focusing on the patient, anticipating their needs, and collaboratively evaluating the relationship over time. Grounded in this relationship, nurses are then able to meet the patient’s relational, psychosocial, and physical needs in an integrated manner—that is, integration of care (Feo et al., 2018). To ensure this, nurses must be competent in addressing these needs while maintaining the nurse–patient relationship (Feo et al., 2018; Kitson et al., 2013; Ranta and Kaunonen, 2024).

Although the provision of fundamental care is part of the broader scope of nurses’ scope of practice (Nursing and Midwifery Council, 2019), it is specifically embedded within the RN’s scope. Scope of practice can be described as “the full spectrum of roles, functions, responsibilities, activities and decision-making capacity which individuals within that profession are educated, competent and authorised to perform” (Malone and Cliffe, 2018, p. 7). For RNs, this includes critical thinking and active participation in clinical decision-making processes, typically structured around the five phases of the nursing process: assessment, analysis, planning and setting goals, implementation, and evaluation of fundamental care needs (Wilkinson, 2012). Non-RNs have a lower-level education than RNs and are not expected to have the same scope of practice (Nursing and Midwifery Council, 2019). However, given reports of task-shifting from RNs to non-RNs and evidence that non-RNs in home-based care settings provide care beyond their training (Saari et al., 2018), it is relevant to examine how clinical decision-making processes and fundamental care are enacted in relation to non-RNs’ scope of practice as well.

The performance of fundamental care activities may involve collaboration with multidisciplinary teams, where nurses operate at dependent, independent, or interdependent levels (Wilkinson, 2012). The nurses’, RNs and non-RNs, level of performance defines the boundaries between care activities carried out independently and those that may be shared or delegated. Moreover, the scope of nursing practice includes adapting fundamental care activities to older people and, when possible, supporting their independence (Henderson, 1964). Such adaptation of the activities’ functional level—whether full, partial, or compensatory—requires knowledge of the older person’s resources, including their strengths and abilities.

Fundamental care is a particularly important part of nurses’ scope of practice in home-based care due to the presence of complex health care needs, which stem from multimorbidity—having two or more chronic conditions—higher age and frequent healthcare utilization (Næss et al., 2017). In home-based care, nurses constitute the largest professional group and have a crucial role and function in shaping both older people’s health and their overall experience of care (Cleland et al., 2021). Today, home-based care has become the primary setting for older people’s care, allowing them to receive care in their own homes rather than in institutional environments, such as nursing homes (Andrade et al., 2017). As a result, nurses in home-based care often work independently in settings that may not be fully optimized for care, while also navigating significant geographical distances between home visits (Helgheim and Sandbaek, 2021; Leverton et al., 2019). Nurses’ ability to enact their scope of practice, including ensuring fundamental care needs, is shaped by a range of contextual modulators, including individual factors—such as nurses’ dexterity, awareness, concentration, and decision-making ability—and organisational factors, such as stress and fatigue among nurses, the physical work environment, safety culture, communication, teamwork, and leadership (Lister et al., 2021). Understanding these modulators is vital, as they influence the quality of care, which is defined as person-centred, safe, effective, efficient, accessible, timely, equitable, and eco-friendly (Lachman et al., 2021). Recent literature reviews indicate that few studies have explored nurses’ scope of practice—particularly decision-making and the presence of contextual modulators—in relation to fundamental care, especially within home-based care settings (Nordaunet et al., 2024; Savoie et al., 2023). Thus, we aimed to explore nurses’ scope of practice, fundamental care, and contextual modulators in relation to older people with complex health care needs in home-based care.

## 2 Methods

### 2.1 Study design

In this study, we employed an exploratory design and collected both numerical data (frequency of observed occurrences of the phenomena of interest) and textual data (descriptive and reflective field notes) through structured direct observations (Fix et al., 2022; Weston et al., 2021).

### 2.2 Research context and setting

Home-based care in the Nordic countries is characterised by public funding and universal accessibility, commonly referred to as the Nordic model (Rostgaard et al., 2022). In Sweden, home-based care has become the primary form of care for older people with complex health care needs (National Board of Health and Welfare, 2025) and is predominantly (approximately 75 %) operated by municipalities (National Board of Health and Welfare, 2022).

Three categories of nursing staff are involved in the provision of home-based care: RNs, nursing assistants, and care assistants. RNs complete a three-year programme at a university or university college, leading to a bachelor's degree in general nursing science and eligibility for professional registration. Specialist education at the master’s level is available in various fields, such as district nursing (Ministry of Education and Research, 1992). In Swedish municipalities, RNs account for approximately 11 % of the total nursing workforce (Swedish Association of Local Authorities and Regions, 2025a, 2025b). Nursing assistants (here referring to non-RNs) are educated at the upper secondary school level. Upon completion, they may apply for licensure (National Board of Health and Welfare, 2023). Care assistants (also non-RNs but not involved in this study) are personnel without formal health-related education. However, the scope of practice for RNs and non-RNs are not identical, and they are regulated under separate legislative acts (Ministry of Social Affairs, 2017, 2025). Assignments for non-RNs are typically determined by social care assessors—municipal officers responsible for evaluating individuals' need for social care—or delegated by RNs, such as tasks involving administration of medication or performing wound care.

### 2.3 Sampling and recruitment strategies

We strived for a maximum variation sample (Onwuegbuzie and Leech, 2007), and the recruitment was conducted in two steps. In the first step, we examined municipalities in Western Sweden, considering factors such as population size and organization to identify meaningful variation. Heads of healthcare in three municipalities—two smaller municipalities with 12,000 and 13,000 inhabitants (sites 2 and 4) and and one larger with 98 000 inhabitants (sites 1 and 3)—accepted an invitation to participate in the study. The four sites (1–4) covered urban, rural, suburban, and semi-urban settings. In all municipalities, non-RNs and RNs worked in separate units (home care units and home nursing units, respectively) that functioned as distinct organizations with separate offices and management. Each site (1–4) comprised one or two home care units and one home nursing unit. At Site 1, the same setting applied to both the home care unit and the home nursing unit, whereas at Sites 2–4, some of the RNs worked primarily in other settings within the municipality. In the second step, the first author visited the units and provided both oral and written information about the study to the nurses during staff meetings. The nurses were eligible for inclusion if they were RNs or nursing assistants (hereafter non-RNs), had been employed at the unit for at least 1 month and gave informed consent. Observations focused on nurses’ scope of practice for older people (65 years or older) diagnosed with two or more chronic conditions, receiving full-time care (defined as three or more home visits per day), and who required care from both RNs and non-RNs.

### 2.4 Data collection and procedure

Direct structured observations (Fix et al., 2022; Weston et al., 2021) were conducted by the first author between April 2024 and November 2024. Direct structured observations were used to capture unique insights into nurses’ scope of practice, its processes, and contextual conditions by watching and listening without being an active participant (Fix et al., 2022). The first author was present for 14 days per site (1–4). To observe as many activities as possible, different strategies were applied for RNs and non-RNs. As the latter had more home visits per shift than RNs, they were observed across four morning shifts (approximately 07:00–16:00) and one evening shift (approximately 15:00–22:00) at each site. RNs, who had fewer home visits a day and had a consultative function during evening shifts, were observed nine morning shifts per site. The observation started when the nurse entered a home and stopped when the nurse walked out. One observation was equivalent to one home visit. The structured direct observations were supported by an observation protocol developed by the research group; see Supplementary Material 1 for further description and operationalisation of items. The protocol encompassed four key areas (Practice Area 1–4) grounded in established nursing concepts. The primary focus was on nurses’ activities related to the Fundamentals of Care framework (Feo et al., 2018), comprising the dimension representing the nurse–patient relationship (five items) together with the dimension integration of care and its sub-dimensions: relational (nine items), psychosocial (eight items) and physical needs (eight items). All elements in those dimensions were included and represented by one item each (Practice Area 1). However, the dimension representing context of care in the Framework (Feo et al., 2018) was not included in this study. Additionally, nurses’ activities related to context-specific psychosocial needs (three items, Practice Area 1) were included, based on the Quality from the Patient’s Perspective framework (From et al., 2015). Further areas addressed nurses’ clinical decision-making (Practice Area 2)—i.e. the positioning of their activities in the phases of the nursing process that is assessment, analysis, planning and setting goals, implementation, and evaluation (Wilkinson, 2012)—as well as the activities’ performance and functional levels (Practice Area 3) (Henderson, 1964; Wilkinson, 2012), and contextual modulators relevant to nurses’ scope of practice (Practice Area 4) (Lister et al., 2021). In total, 55 items were included. Each item was observed and documented regarding whether it was verbal or observable. The protocol also included prompts for recording key details of the home visit, such as date, time, and individuals involved. Additionally, space was provided for descriptive and reflexive field notes. The field notes were continuously labelled with their corresponding observation item. Before each observation, the participant was asked to state the primary reason for the home visit, which was then recorded in the observation protocol. The protocol was piloted and tested for inter-rater reliability at a clinical skills centre, and its layout was subsequently revised based on the evaluation of the pilot test.

### 2.5 Data analysis

Numerical data were analysed using descriptive statistics, with analyses conducted in IBM SPSS Statistics and Microsoft Excel. Means and ranges were calculated for continuous variables. For nominal-level data, the dichotomous variables (‘yes’/‘no’) were presented as frequencies, proportions, and prevalence (Field, 2024). Thus, we focused on what nurses did, not on what older people required. To account for variation in the number of observations per participant, the arithmetic mean for each dichotomous variable was calculated at the individual level (0 to 1), reflecting the prevalence with which each participant performed the activity across observed occasions. Additionally, textual data—i.e., field notes labelled with their corresponding observation item—were incorporated into the results to contextualise the numerical findings.

### 2.6 Ethical considerations

We adhered to established ethical guidelines (World Medical Association, 2025) and received approval from the Swedish Ethical Review Authority (Dnr 2023–07,971-01). All participants—including RNs and non-RNs—received both verbal and written information prior to providing written informed consent. They were informed of their right to withdraw from the study at any time without giving a reason. Older people were also provided with verbal and written information and asked for consent prior to each observation. All data were systematically coded, securely transferred, and stored in accordance with data protection regulations. Only individuals with approved authorization had access to the data.

## 3 Results

In total, 230 observations were conducted, involving 23 RNs and 23 non-RNs. Each group consisted of 21 women and two men. The RNs were aged 26–59 years (mean = 41; median = 39.5), while the non-RNs were aged 19–68 years (mean = 41; median = 44). Regarding professional experience, RNs had between 0.5 and 35 years in the profession (mean = 12.1; median = 8), and non-RNs between 0.5 and 36 years (mean = 12.8; median = 7). The predominant social context involved older people living alone in apartments, although living in houses was more common at sites 2 and 4 than at sites 1 and 3. Cohabiting older people were more frequently observed at site 1 compared to the other sites. Participants working in pairs were more frequently observed at sites 1 and 2 than at sites 3 and 4 (Table 1).

**Table 1:** Descriptive characteristics of the observations, overall and by site.

**Characteristics**	**Observations** **sites 1–4**	**Observations** **site 1**	**Observations** **site 2**	**Observations** **site 3**	**Observations** **site 4**
Observations *N* ( %)	230	59(26)	69(30)	44(19)	58(25)
Older people receiving care *n* ( %)	109	23(21)	22(20)	23(21)	41(38)
*Place of observation n* ( *%*)					
-Apartment	155(67)	30(51)	53(77)	43(98)	29(50)
-House	75(33)	29(49)	16(23)	1(2)	29(50)
*Social context of observation n* ( *%*)					
-Older people living alone	172(75)	36(61)	50(72)	39(89)	47(81)
-Older people living with spouse	49(21)	19(32)	14(20)	5(11)	11(19)
-Older people living with adult child or intermittently residing with a partner	9(4)	4(7)	5(7)	-	-
*Length of observations n* ( *%*)					
=1–15 min	98(44)	25(42)	37(54)	19(43)	17(29)
=16–30 min	80(35)	20(34)	21(30)	12(27)	27(45)
=31–45 min	28(12)	3(5)	5(7)	8(18)	12(21)
=46–60 min	10(4)	3(5)	4(6)	1(2.)	2(3)
=61–115 min	14(6)	8(14)	2(3)	4(9)	—
*Days observed n* ( *%*)					
-Monday – Friday	220(96)	49(83)	69(100)	44(100)	58(100)
-Weekends/bank holidays	10(4)	10(17)	—	—	—
*Shifts observed n* ( *%*)					
-Morning shift	155(67)	36(61)	51(74)	31(70)	37(64)
-Afternoon shift	75(33)	23(39)	18(26)	13(30)	21(36)
*Activities nurses engaged in n (%)*					
-Activities related to the establishment and maintenance of the nurse-patient relationship	796(26)	210(25)	228(26)	141(26)	217(28)
-Activities related to relational needs	979(32)	259(31)	262(30)	181(33)	277(35)
-Activities related to psychosocial needs	679(22)	206(24)	203(23)	110(20)	160(20)
-Activities related to physical needs	588(19)	172(20)	175(20)	112(21)	129(16)
*Nursing staff part of observations n* ( *%)*					
-Registered nurses	89(39)	22(37)	26(38)	17(39)	24(41)
-Non-registered nurses	141(61)	37(63)	43(62)	27(61)	34(59)
*Team composition in observations n* ( *%*)					
-Working alone	198(86)	49(83)	56(81)	40(91)	53(91)
-Working in pairs	32(14)	10(17)	13(19)	4(9)	5(9)
*Participants n* ( *%*)					
-Registered nurses	23(50)	3(38)	8(62)	5(42)	7(54)
-Non-registered nurses	23(50)	5(63)	5(39)	7(58)	6(46)

Notes**:***N* = total number; *n* = *a* subset of the total number.

In 94 % of home visits, participants reported that their main purpose was to address physical needs. On average, the participants engaged in 13.23 activities related to fundamental care needs per observation. In the following section, the activities are presented based on their position in the Framework (Feo et al., 2018).

### 3.1 Activities within the dimension of nurse–patient relationship

During the 230 observations, participants engaged in 796 activities related to building and maintaining the nurse-patient relationship (Table 2). Observed activities were primarily associated with building trust, being focused, having knowledge about the older people as individuals, and anticipating their needs. Both RNs and non-RNs engaged in building and maintaining the relationship in all (100 %) of the observations. However, activities involving the evaluation of the relationship through dialogue with the patient were observed in 0.7 % of observations (*n* = 1).

**Table 2:** Overview of observed activities’ (*N* = 3042) and contextual modulators (*N* = 1610).

Fundamentals of Care (Feo et al, 2017)	**Observations**	**Observations**	**Observations**	**Observations**	**Observations**	**Observations**
	**sites 1-4**	**sites 1-4**	**sites 1-4**	**sites 1-4**	**sites 1-4**	**sites 1-4**
	**RN and non-RN**	**RN**	Non-RN	**RN and non-RN**	**RN**	**Non-RN**
	**(*N* = 230)**	**(*n* = 89)**	**(*n* = 141)**	**(*N* = 230)**	**(*n* = 89)**	**(*n* = 141)**
	Frequency *n* and proportion (%)			Prevalence* (% of observations)		
**Observed activities within the dimension of nurse-patient relationship (*N*)**	796	310	486	100	100	100
-Build trust	218(27)	86(28)	132(27)	96	97	96
-Focus on the older people	210(26)	85(27)	125(26)	96	98	93
-Anticipate needs	174(22)	67(22)	107(22)	73	72	74
-Know or getting to know the older people	193(24)	71(23)	122(25)	80	76	83
-Evaluate the relation	1(<1)	1(<1)	0(0)	<1	1	0
**Observed activities within the dimension of integration of care (*N*)**	2246	823	1423	100	100	100
*Observed activities within the sub-dimension of relational needs* (*N*)	979	380	599	95	99	91
-Being empathic	181(18)	67(18)	114(19)	81	81	80
-Helping older people to cope	13(1)	7(2)	6(1)	8	10	6
-Engaging with older people	181(18)	68(18)	113(19)	81	78	83
-Supporting and involving families	44(4)	24(6)	20(3)	21	28	14
-Working with older people to set goals	1(<1)	1(<1)	0(0)	1	1	0
-Active listening	193(20)	80(21)	113(19)	89	94	84
-Helping older people to stay calm	24(2)	7(2)	17(3)	8	9	7
-Being compassionate	154(16)	54(14)	100(17)	71	69	74
-Being present	188(19)	72(19)	116(19)	86	87	84
*Observed activities within the sub-dimension of psychosocial needs* (*N*)	679	256	423	100	100	100
-Communication adjusted to the older people	220(32)	85(33)	135(32)	97	98	97
-Being involved and informed	217(32)	80(31)	137(32)	95	93	98
-Respect	41(6)	14(5)	27(6)	19	16	21
-Education and information	46(7)	33(13)	13(3)	26	42	9
-Having values and beliefs considered and respected	52(8)	16(6)	36(9)	22	20	23
-Dignity	23(3)	8(3)	15(4)	8	8	8
-Emotional wellbeing	17(3)	9(4)	8(2)	10	13	8
-Privacy	14(2)	4(2)	10(2)	10	5	15
-Help to get outside^a^	10(1)	4(2)	6(1)	7	6	8
-Help to perform meaningful activities^a^	13(2)	1(<1)	12(3)	4	<1	7
-Access to personal belongings^a^	26(4)	2(1)	24(6)	9	<1	18
*Observed activities within the sub-dimension of physical needs* (*N*)	588	187	401	97	100	95
-Rest and sleep	24(4)	6(3)	18(4)	9	8	11
-Personal cleaning and dressing	67(11)	13(7)	54(13)	24	15.9	32
-Medication management e.g., preparation and administration	121(21)	33(18)	88(22)	54	39	68
-Toileting	72(12)	17(9)	55(14)	27	21	33
-Eat and drink	87(15)	14(7)	73(18)	35	20	50
-Comfort including vital parameters, pain, breathing, wound care, and positioning	114(19)	79(42)	35(9)	57	91	24
-Safety e.g., risk assessment, infection prevention	23(4)	9(5)	14(3)	13	15	11
-Mobility	80(14)	16(9)	64(16)	34	23	45

**Contextual modulators (Lister et al., 2021)**				**Observations**	**Observations**	**Observations**
				**sites 1-4**	**sites 1-4**	**sites 1-4**
				**RN and non-RN**	**RN**	**Non-RN**
				**(*N* = 230)**	**(*n* = 89)**	**(*n* = 141)**
				Prevalence* (% of observations)		
*Observed direct (individual) modulators*				100	100	100
-Mental and physical dexterity/Yes				93	93	93
-Ability to take note and assess the situation/Yes				81	87	75
-Ability to concentrate during activities and not being interrupted/Yes				61	54	69
-Ability to make decisions in a situation/Yes				92	88	96
*Observed potential (organisational) modulators*				100	100	100
-No stress verbalised /Yes				86	92	81
-No fatigue verbalised/Yes				86	86	87
-Identifies and prevent risks/Yes				41	66	17
-Information transfer takes place between staff/Yes				32	38	27
-Ability to manage and lead nursing care/Yes				43	72	14
-Work environment positively supports nurses in tasks/Yes				54	40	68
-Co-operation between staff with different responsibilities/Yes				25	44	5

Notes: ^a^Context-specific activities (From et al. 2015).

*N* = total number; *n* = *a* subset of the total number.

RN = Registered nurse. Non-RN = non-registered nurse.

### 3.2 Activities within the dimension of integration of care

In total, participants engaged in 2246 activities representing the dimension integration of care—that is, addressing psychosocial, relational, and physical needs. Most of these activities addressed relational needs (44 %), followed by psychosocial (30 %) and physical needs (26 %). A greater proportion of non-RNs observed activities were related to physical needs compared to those of RNs (28 % and 23 %, respectively), whereas RNs appeared to be more engaged in activities addressing relational needs than non-RNs (46 % and 42 %, respectively) (Table 2).

Participants addressed relational needs by engaging in activities that demonstrated active listening, presence, engagement, empathy, and compassion. However, activities focused on helping older people cope and remain calm were observed less frequently, accounting for 1 % and 2 % of the relational activities, respectively. The least recorded activity was goal setting with older people, which was documented in only one observation (*n* = 1). RNs were observed to be more engaged in involving families (28 % of the observations) than non-RNs (14 % of the observations).

Participants’ activities to address older people’s psychosocial needs primarily involved adjusting communication and keeping older people informed and involved. However, participants were observed to be less engaged in activities targeting older people’s dignity, privacy, and emotional well-being (accounting for 3 %, 3 %, and 2 % of the psychosocial activities, respectively). Regarding differences between RNs and non-RNs, the observations indicated that RNs were more engaged in education and information (42 % of the observations) compared to non-RNs (9 % of the observations). In contrast, non-RNs were observed to be more engaged in activities related to context-specific needs (From et al., 2015) than RNs, especially regarding access to personal belongings (18 % and <1 % of the observations, respectively) and participation in meaningful activities (7 % and <1 % of the observations, respectively), exemplified in the fieldnotes:

*The non-RN helped the older woman turn on the TV. She knew what kind of TV shows the woman liked and turned on a children’s program for her* [Psychosocial needs: help to perform meaningful activities] (Observation number 139)

Participants’ activities related to older people’s physical needs were focused on, presented in order of proportional size: medication management (e.g., preparation and administration); comfort (including vital parameters, pain, breathing, wound care, and positioning); eating and drinking; mobility; toileting; and personal cleaning and dressing. This is exemplified in the fieldnotes:

*The non-RN gave eye drops and injected insulin*. [Physical needs: medication management] (Observation number 127)

Activities related to safety (e.g., risk assessment, infection prevention) were the least performed, accounting for 4 % of the activities targeting physical needs. RNs were observed to be more engaged in activities related to comfort (including vital parameters, pain, breathing, wound care, and positioning) than non-RNs, recorded in 91 % and 24 % of the observations, respectively. However, all physical needs—except for comfort and safety—were more often observed to be addressed by non-RNs than by RNs.

### 3.3 Activities within the clinical decision-making process

**Fig. 2:**
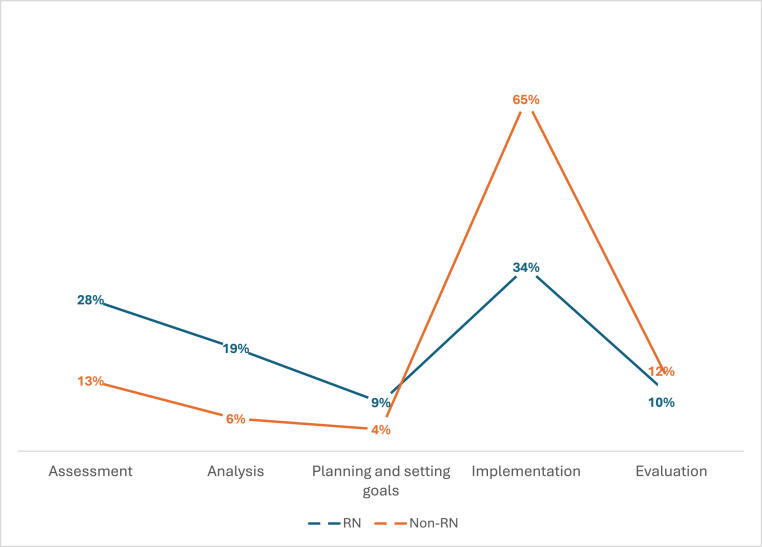
Distribution of observed engagement of RNs and non-RNs in activities addressing physical needs across the five phases of the nursing process; i.e., the clinical decision-making process. Notes: RN = Registered Nurse. Non-RN = Non-Registered Nurse.

Clinical decision-making among participants, operationalised through the phases of the nursing process, was observed in relation to activities targeting physical needs. The observations revealed that a single activity addressing a specific need could be situated within multiple phases of the process during one observation. Participants demonstrated verbal or observable engagement in clinical decision-making 978 times during the observations. The engagement was proportionally distributed across the phases as follows: implementation (52 %), assessment (19 %), analysis (12 %), evaluation (11 %), and planning and setting goals (6 %). Both RNs and non-RNs were involved in all phases of the clinical decision-making process (Fig. 2). However, while non-RNs primarily contributed during the implementation phase, RNs were predominantly observed to be engaged in both the assessment and implementation phases.

**Fig. 3:**
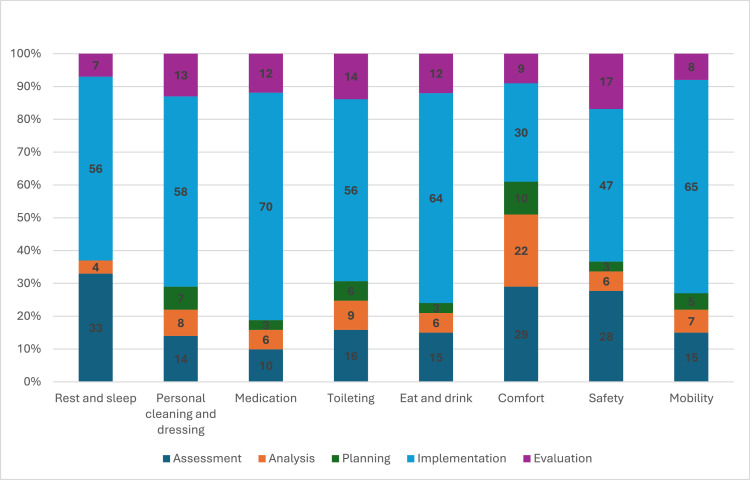
Proportional distribution of activities addressing physical needs across the five phases of the nursing process; i.e., the clinical decision-making process.

For all activities related to physical needs, participants’ engagement in clinical decision-making tended to be concentrated in the implementation phase, representing up to 70 % of the engagement (Fig. 3). In contrast, participants’ engagement in clinical decision-making concerning comfort showed a more proportional distribution across the process: assessment (29 %), analysis (22 %), planning and setting goals (10 %), implementation (30 %), and evaluation (9 %). Other activities addressing physical needs were generally sparse in the analysis and planning phases, and no engagement (*n* = 0) related to rest and sleep was observed in the planning phase.

### 3.4 Activities performance- and functional level

Participants’ activities related to physical needs were also observed based on their level of performance; i.e., professional autonomy: dependent (initiated by another profession), independent (initiated within their own profession), or collaborative (initiated by a multidisciplinary team) (Wilkinson, 2012). Non-RNs were observed to be more engaged in dependent activities (92 % of the observations) than RNs (32 % of the observations). Conversely, non-RNs were less engaged in independent activities than RNs (26 % and 63 % of the observations, respectively). For both professions, independent activities were predominantly performed in relation to comfort. Additionally, RNs participated in collaborative activities in 16 % of the observations, primarily related to comfort. In contrast, non-RNs were not observed to engage in any collaborative activities.

Observations related to the activity’s functional level showed that partially-compensating care—activities involving input or participation from older people—occurred in 87 % of the observations, each involving either an RN or a non-RN. Older people acted as equal partners during activities in 15 % of the observations, most commonly in relation to personal cleaning and dressing (6 % of the observations). In contrast, compensatory care—activities performed without input or involvement from older people—was documented in 18 % of the observations, most commonly in activities related to medication management (6 % of the observations).

### 3.5 Contextual modulators in nurses’ scope of practice

Contextual modulators were present in all observations (Table 2). Among individual modulators, dexterity (93 % of the observations) and decision-making (92 % of the observations) were the most observed. However, the ability to concentrate and not being interrupted was less common, occurring in 69 % of the observations focusing on non-RNs and 54 % of the observations focusing on RNs. These interruptions were described in fieldnotes, as in this example:

*The RN was providing wound care when the telephone rang. The RN answered the phone and gave instructions to a non-RN about medication administration, then hung up and continued with wound care* [Individual modulator: Ability to concentrate] (Observation number 195).

Regarding organizational modulators, RNs were observed to be more engaged in safety culture—e.g., hand hygiene—co-operation, and leadership than non-RNs, whereas non-RNs were observed to have a more supportive work environment than RNs.

## 4 Discussion

We aimed to explore nurses’ scope of practice, fundamental care, and contextual modulators in relation to older people with complex health care needs within the Swedish home-based care context. There has been a reported lack of research investigating fundamental care in out-of-hospital contexts (Savoie et al., 2023), and, to our knowledge, we are the first to explore nurses’ scope of practice using the Fundamentals of Care Framework (Feo et al., 2018) in a home-based care context. We revealed that participants’ activities during home visits often extended beyond the initial purpose, which in 94 % of the observations was described as to assess or address a physical need. An initial focus on a specific need frequently evolved into a wider range of activities addressing multiple dimensions of care, reflected in an average of 13.22 activities per home visit. This expansion of activities illustrates the complexity of nurses’ scope of practice in home-based care and contributes to the growing body of knowledge on the framework’s relevance for practice (Pene et al., 2025).

We have demonstrated that nurses engage in a wide range of activities reflecting the dimensions of integration of care (i.e., addressing relational, psychosocial, and physical needs) and the nurse–patient relationship. Moreover, several context-specific activities were identified, informed by items from the Quality of Care from the Patient’s Perspective framework (From et al., 2015). These findings suggest that, while the Fundamentals of Care Framework (Feo et al., 2018) provides a valuable foundation for practice and research, it may benefit from further refinement or contextual adaptation to fully capture the complexity of nurses’ scope of practice in home-based care.

We revealed that participants were involved in a variety of activities aimed at establishing and maintaining the nurse–patient relationship. However, collaborative evaluation of this relationship was observed in only 0.7 % of the observations—an infrequent occurrence that, although lower, aligns with findings from a study conducted in Norwegian nursing homes (Nordaunet et al., 2025). Furthermore, evaluation of the nurse–patient relationship is rarely addressed in existing research (Feo et al., 2022), highlighting a gap in both scholarly literature and clinical practice. We argue that the quality of the nurse–patient relationship and older people’s participation in fundamental care could be strengthened through the implementation of structured, collaborative evaluation processes.

Both RNs and non-RNs demonstrated verbal or observable engagement in the phases of the clinical decision-making process, although the engagement of RNs appeared to be more comprehensive. This is arguably expected, given that assessment, analysis, planning and setting goals, and evaluation of care are often viewed as key elements that differentiate the RNs’ scope from that of non-RNs’ (Nursing and Midwifery Council, 2019). In the present study, non-RNs were primarily engaged during the implementation phase, functioning as the “operating arm” (Richards and Borglin, 2019) for activities coordinated by RNs or social care assessors. Although clinical decision-making is not typically considered to fall within the scope of practice for non-RNs (Nursing and Midwifery Council, 2019), over 30 % of their engagement in clinical decision-making was situated within the assessment, analysis, planning and setting goals, or evaluation phases. Based on these findings, it remains unclear whether non-RNs’ involvement beyond the implementation phase was formally within their professional remit. Further research is warranted to explore the nature of non-RNs’ clinical decision-making and clinical reasoning in the context of home-based care. Regarding RNs’ involvement in the clinical decision-making process, we suggest that they may experience difficulties in consistently implementing all phases of the decision-making process. Notably, their observable practice often involved assessment and implementation, rather than analysis, planning and setting goals, and evaluation. Lack of evaluation is consistent with previous research in nursing homes (Nordaunet et al., 2025), which highlighted similar challenges. A lack of sustained engagement analysis, planning and setting goals, and evaluation contributes to fragmented care delivery and adversely affects patient outcomes (Guerrero, 2019; Levett-Jones et al., 2010). Providing RNs with appropriate tools—such as targeted education (Ghodsi Astan et al., 2022) and adequate resources—may strengthen their ability to implement all phases of the decision-making process and thereby improve the overall quality of care.

Performing independent activities (Wilkinson, 2012)—defined as initiating the activity—alongside clinical decision-making is a hallmark of the RN’s scope of practice and is not typically expected of non-RNs (Nursing and Midwifery Council, 2019). Nevertheless, we found that non-RNs independently initiated activities addressing older people's fundamental care needs in 26 % of the observations. Autonomy in responding to fundamental care needs may be a valuable resource for non-RNs (Huang et al., 2025), yet it also entails risks when situations exceed their competence (Saari et al., 2018). While we did not determine whether the activities initiated by non-RNs were within their professional boundaries, it underscores the need for further research into non-RNs’ autonomy in home-based care.

Nurses’ scope of practice in relation to fundamental care appears to be related to various contextual modulators. Observational data highlighted frequent interruptions during home visits and an unsupportive physical work environment, particularly in the case of RNs. Interruptions may stem from complex and multifactorial causes and have, in previous studies, been associated with negative impacts on patient safety (Wang et al., 2021). However, we did not determine whether, or in what ways, the observed contextual modulators affected the delivery of fundamental care. Further research is therefore needed. In addition to the time cost they entail, both interruptions and an unsupportive work environment may contribute to increased stress, workload, and nurses’ intentions to leave the profession (Bahlman‐van Ooijen et al., 2023). To promote nurses’ wellbeing and support their role and function in fundamental care to older people in home-based care, healthcare organisations should consider addressing contextual modulators as part of quality improvement efforts.

## 5 Methodological considerations

A key strength of this study is its exploratory design, which enabled in-depth insight into nurses’ everyday scope of practice, fundamental care, and contextual modulators in home-based care. Structured direct observations (Fix et al., 2022; Weston et al., 2021), guided by a piloted protocol grounded in the Fundamentals of Care Framework (Feo et al., 2018) and other established nursing concepts, facilitated a nuanced understanding of the phenomenon under investigation. The combination of numerical and textual data enhanced the study’s credibility. While the framework provided a robust foundation for observation, its application in home-based care required contextual adaptation to capture the specific nuances of fundamental care in this setting. The large number of observations conducted across multiple sites over an extended period likely reduced the risk of biased data. However, the findings reflect only a small segment of the Swedish home-based care context, which may limit their transferability. Observation strategies and durations were tailored to the working conditions of non-RNs and RNs. Despite these adjustments, fewer observations were conducted with RNs than with non-RNs, which may have affected the scope and depth of the activities captured. Data collection was conducted by a single researcher—an RN with experience in home-based care. While her professional background may have influenced the process, it also enabled a nuanced understanding of clinical practice. Regarding validity, the observation protocol was based on established nursing concepts and included clear operational definitions, ensuring consistency in data collection. However, interpretation during analysis may have been influenced by situational factors. To reduce this risk, the observer maintained a neutral stance, although complete objectivity is inherently challenging. The observations were non-participatory, with the observer remaining silent and in the background during home visits. The nurses’ awareness of being observed may have affected their behaviour and activities, but they were informed that we aimed to describe their scope of practice rather than evaluate performance.

## 6 Conclusion

We aimed to explore nurses’ scope of practice, fundamental care, and contextual modulators in relation to older people with complex health care needs in home-based care. We are among the first to explore nurses’ scope of practice in this setting using the Fundamentals of Care Framework as a conceptual foundation. We have shown that nurses undertake a broad range of activities to meet older people's fundamental care needs. Clinical decision-making among RNs was largely characterised by assessment and implementation, with limited involvement in the full continuum of the nursing process. We revealed that non-RNs were active in the clinical decision-making process, and they also independently initiated activities, indicating ongoing task-shifting from RNs to non-RNs. Furthermore, nurses’ scope of practice was observed alongside several contextual modulators, reflecting the multifaceted nature of home-based care. We suggest that these findings may inform the future development of targeted interventions or Models of Care grounded in the framework specifically tailored for home-based care and taking contextual modulators into account

## 7 Disclosure

This project is part of the Nordic programmatic research collaboration ‘Continuity and Quality of Care’ at Karlstad University, Sweden, and Lovisenberg Diaconal University College, Norway. This research report is the second study of a PhD-project using multi-methods focusing on nurses’ scope of practice, fundamental care, older people, and the context of home-based care.

## Funding

No external funding. The PhD-project is funded by Karlstad University.

## Data availability

The raw data supporting the findings of this study are not publicly available due to confidentiality and ethical restrictions, that prohibit data sharing. However, the developed and tested observational protocol is available upon reasonable request from the corresponding author.

## Declaration of generative AI in scientific writing

Language refinement and sentence structure improvements in the final manuscript were supported by Microsoft Copilot, a GDPR-compliant AI tool based on ChatGPT. The tool was used solely for linguistic assistance; all content and interpretations are the responsibility of the authors.

## CRediT authorship contribution statement

**Karin Sandberg:** Writing – review & editing, Writing – original draft, Visualization, Software, Project administration, Methodology, Investigation, Formal analysis, Data curation, Conceptualization. **Anna Josse Eklund:** Writing – review & editing, Supervision, Methodology, Formal analysis, Conceptualization. **Gunilla Borglin:** Writing – review & editing, Supervision, Methodology, Conceptualization. **Edith Roth Gjevjon:** Writing – review & editing, Supervision, Conceptualization. **Cecilia Olsson:** Writing – review & editing, Supervision, Project administration, Methodology, Formal analysis, Conceptualization.

## Declaration of competing interest

The authors declare that they have no known competing financial interests or personal relationships that could have influenced the work reported in this paper.

## Appendix Supplementary materials

Image, application 1
