# Supplementary material for: Nurses’ scope of practice and fundamental care in relation to older people: An exploratory home-based study
Source: Int J Nurs Stud Adv. 2026 Jan 26;10:100492. doi: 10.1016/j.ijnsa.2026.100492 (PMC12905735; doi:10.1016/j.ijnsa.2026.100492)
Supplement: Supplementary file 1 [file mmc1.docx]

Table 1. Overview content of the observational protocol.

| **Observational protocol area** | **Operationalisation and description** |
| --- | --- |
| *Practice Area 1* | *Nurses’ activities* *corresponding to the Fundamental of Care Framework (Feo et al., 2018)* |
| *(Units of observation)* | Activities related to *the nurse-patient relationship (5 items):* |
|  | *Build trust -* Develops relationship. Introduce yourself, friendly |
|  | *Focus on the older people* - Eye contact, body language, interprets signals |
|  | *Anticipate needs* - Prior experience used |
|  | *Know or getting to know the older people* - Knowledge of health history and personal preferences |
|  | *Evaluate the relation* - Evaluate the relationship in dialogue with the patient |
|  | *Activities related to relational needs (9 items)* |
|  | *Being emphatic* - Understands the patient's situation |
|  | *Helping older people to cope -* Supported in finding/maintaining coping strategies |
|  | *Engaging with older people -* Sees the patient as an individual, e.g., small talk |
|  | *Supporting and involving families -* Provide support to family, promote their participation |
|  | *Working with older people to set goals -* Sets goals together with the patient |
|  | *Active listening -* Showing responsiveness |
|  | *Helping older people to stay calm -* Helping older people to find and maintain calm |
|  | *Being compassionate -* Show care for older people |
|  | *Being present -* Be physically and mentally with the older people |
|  | Activities related to *psychosocial needs (8 items):* |
|  | *Communication adjusted to the older people* - Oral and written. Adapted to the older people. |
|  | *Being involved and informed -* Be involved, be informed about the care |
|  | *Respect -* Respected as a person and not overruled. |
|  | *Education and information -* Get education and information |
|  | *Dignity -* Do not embarrass/offend e.g. in case of lower hygiene |
|  | *Having values and beliefs considered and respected -* Consideration of values, beliefs, feelings, preferences, opportunity to make own choices |
|  | *Emotional well-being -* Mental well-being, e.g. anxiety, stress, depression |
|  | *Privacy -* Right to privacy. Do not speak over the person’s head |
|  | *Activities related to physical needs (8 items):* |
|  | *Rest and sleep* - Adapt/promote opportunities for sleep and rest |
|  | *Personal cleansing and dressing* - Upper-lower hygiene, oral care, showering, clothing |
|  | *Medication management -* Handling of medicines, management, preparation, administration. |
|  | *Toileting -* Empty bowels and bladder |
|  | *Eat and drink -* Food and drinks that are adapted to the person |
|  | *Comfort – Physical wellbeing, vital* parameters, pain, breathing, positioning, wound care |
|  | *Safety -* Risk assessments, prevent infections |
|  | *Mobility -* Helping older people to move |
|  | *Activities related to context specific psychosocial needs* (From et al., 2015) that is: Help to get outside, Help to perform meaningful activities, Access to personal belonging. |
| *Practice Area 2* | *The decision-making process* operationalised as the nursing process (Wilkinson, 2011) that is: Assessment, Analysis and diagnosis, Planning and setting goals, Implementation, and Evaluation. |
| *Practice Area 3* | The nursing activities’ *performance level* according to Henderson (1997), that is, the compensatory level: Compensating care, partially compensating care or collaborative care. |
|  | The nurses’ *level of professional independence* – autonomy – in the activity, that is, dependent, interdependent or independent (Wilkinson, 2011). |
| *Practice Area 4* | Individual and organisational *modulators of context* such as practical skill, awareness, stress, work environment and leadership style (Lister et al., 2020). |
